# Field-Deployable Computer Vision Wood Identification of Peruvian Timbers

**DOI:** 10.3389/fpls.2021.647515

**Published:** 2021-06-02

**Authors:** Prabu Ravindran, Frank C. Owens, Adam C. Wade, Patricia Vega, Rolando Montenegro, Rubin Shmulsky, Alex C. Wiedenhoeft

**Affiliations:** ^1^Department of Botany, University of Wisconsin, Madison, WI, United States; ^2^Forest Products Laboratory, Center for Wood Anatomy Research, United States Department of Agriculture Forest Service, Madison, WI, United States; ^3^Department of Sustainable Bioproducts, Mississippi State University, Starkville, MS, United States; ^4^Department of Wood Science and Engineering, Oregon State University, Corvallis, OR, United States; ^5^Department of Wood Industry, Universidad Nacional Agraria La Molina, Lima, Peru; ^6^Department of Forestry and Natural Resources, Purdue University, West Lafayette, IN, United States; ^7^Departamento de Ciências Biolôgicas (Botânica), Universidade Estadual Paulista—Botucatu, São Paulo, Brazil

**Keywords:** XyloTron, wood identification, illegal logging and timber trade, computer vision, machine learning, deep learning

## Abstract

Illegal logging is a major threat to forests in Peru, in the Amazon more broadly, and in the tropics globally. In Peru alone, more than two thirds of logging concessions showed unauthorized tree harvesting in natural protected areas and indigenous territories, and in 2016 more than half of exported lumber was of illegal origin. To help combat illegal logging and support legal timber trade in Peru we trained a convolutional neural network using transfer learning on images obtained from specimens in six xylaria using the open source, field-deployable XyloTron platform, for the classification of 228 Peruvian species into 24 anatomically informed and contextually relevant classes. The trained models achieved accuracies of 97% for five-fold cross validation, and 86.5 and 92.4% for top-1 and top-2 classification, respectively, on unique independent specimens from a xylarium that did not contribute training data. These results are the first multi-site, multi-user, multi-system-instantiation study for a national scale, computer vision wood identification system evaluated on independent scientific wood specimens. We demonstrate system readiness for evaluation in real-world field screening scenarios using this accurate, affordable, and scalable technology for monitoring, incentivizing, and monetizing legal and sustainable wood value chains.

## Introduction

State-owned Amazonian forests cover 60% of the total area of Peru with over 15.3 million hectares of the Amazon forest being classified as natural protected areas (SERNANP, 2020) and the rest supporting diverse modes of managed production (e.g., 11 million hectares designated as Forest Logging Concessions; Kometter, 2019). However more than 68% of supervised logging concessions showed unauthorized tree harvesting from natural protected areas and indigenous territories (Finer et al., 2014), and in 2016 alone at least 58% of exported lumber was of illegal origin (SBS and GIZ, 2018). According to official data, over the past decade the volume of wood produced from illegally logged trees increased from 1.15 to 1.8 million cubic meters per annum (OSINFOR, 2015—onward).

For the last twenty years Peru has been building governance infrastructure to achieve sustainability of its forest products, facilitated by national and international policies (Office of the US Trade Representative, 2006; SERFOR, 2015) to improve the monitoring and regulation of the forest products supply chain. Oversight of this monitoring is conducted at inspection stations by government authorities such as the National Forestry and Wildlife Service, the Supervisory Agency for Forest and Wildlife Resources, the Regional Offices of Forests and Wildlife, and the National Customs Superintendency of Peru. Rapid field identification of wood can help efficiently establish due cause for further investigation (UNODC, 2016) at these inspection stations when officials are confronted with falsified documentation. In contrast to plant identification, which is based on common botanical structures (i.e., flowers, fruits, leaves), conventional wood identification is dependent on recognizing anatomical patterns in wood and comparing them to reference descriptions or specimens. Such identifications are best performed by highly trained wood anatomists with substantial training in forensic wood identification, and are typically conducted in a laboratory, which does not meet the needs for rapid field screening at the inspection stations.

In the larger Amazonian context, two notable initiatives that enable human-based wood identification are: a mobile phone-based identification key that enables humans to identify 157 species (Gontijo et al., 2017), and the development of electronic identification keys as part of the Brazil-Colombia Amazon Cooperation Treaty Organization (OTCA, 2018). The knowledge of wood anatomical characteristics of Peruvian species conveyed in academic publications (Acevedo and Kikata, 1994; Chavesta, 2015, 2018) and industry guides/manuals (Rodriguez and Sibille, 1996; Gonzales, 2008; Ugarte and Mori, 2018) have not yet been encapsulated in similar solutions and adopted for widespread human-based wood identification in Peru. The development and uptake of these solutions at the national level in Peru has been challenging, in part, due to limited institutional wood forensics capacity, limited opportunities (university courses and infrequent workshops) for human expertise development, and mostly localized access to xylaria for comparative forensic work (the largest Peruvian xylarium, with around 8,500 samples, is housed in the National Agrarian University, Lima). It should be noted that these approaches emphasize or depend on developing human-based expertise.

To remove the need for extensive human expertise and to enable officials with only a modicum of training to identify wood, computer vision-based approaches (Khalid et al., 2008; Ravindran et al., 2018) have been explored for automated wood identification. Souza et al. (2020) and de Andrade et al. (2020) used machine learning for macroscopic image-based identification for woods of 21 and 46 Brazilian species, respectively. Apolinario et al. (2018) used a convolutional neural network (CNN) for identification of 7 commercial Peruvian timber species using a portable microscope. Recently, the open source XyloTron system (Ravindran et al., 2020), was used to demonstrate a field deployable computer vision wood identification model for fourteen commercial Colombian woods by Arévalo et al. (2021). Among these works, it should be noted that XyloTrons have been shown to have comparable/better accuracy than expensive mass spectrometric methods (Ravindran and Wiedenhoeft, 2020), have been deployed for charcoal identification across the European Union in partnership with the Forest Stewardship Council (as noted in Wiedenhoeft, 2020), and, critically, have been ***field-tested*** for wood identification in Ghana (Ravindran et al., 2019). This field testing of a machine learning model on wholly new specimens, ideally by distinct users and using distinct instantiations of the system, especially at the scale undertaken in this work, is lacking in virtually all forensic wood identification literature, regardless of the modality, technique, or the taxa studied.

In this study, we train 24 class (228 taxa grouped into anatomically informed classes representing 57% by volume of the commercially harvested roundwood and 66% by volume of the sawn wood produced in 2019 in Peru; SERFOR, 2020) CNN based computer vision identification models of Peruvian commercial woods for the XyloTron. We use wood specimens from the MADw, SJRw, BCTw, BOFw, Tw, and FORIGw xylaria to develop five-fold cross-validated models and then train a field model using the same hyperparameter values. The field model was trained by incorporating all the images and specimens used in the cross-validation analysis but was evaluated on completely different specimens from the PACw xylarium, using different hardware and different operators. Performance evaluation of an automated wood identification system requires expert verification of each specimen identified by the system and can be logistically challenging. Our approach using verified, mutually exclusive specimens from distinct xylaria during the training and testing phases serves as a practical surrogate for field evaluation (a first step toward real-world field deployment) and provides a useful measure of the generalization capability of the identification system. To the best of our knowledge this is the first, large-scale study of Peruvian commercial timber identification using distinct instantiations of a computer vision identification system, in this case, the XyloTron.

## Materials and Methods

### Species Selection

The 24 Peruvian woods selected for this study represent 57% by volume of the commercially harvested roundwood and 66% by volume of the sawn wood produced in 2019 in Peru (SERFOR, 2020) and are listed in Supplementary Material 1. Because wood anatomy is typically accurate only to the genus level (Gasson, 2011) and given that the XyloTron operates on macroscopic anatomical variation, we included a range of wood anatomically appropriate, congeneric, Amazonian species and restricted data collection to the transverse surface of the specimens (e.g., congeneric species that are differentiable only from the tangential surface are clubbed into the same class here).

### Sample Preparation

The transverse surface of 1,419 wood specimens from seven xylaria (Table 1) were polished by sanding with progressively finer-grit sandpapers (240, 400, 600, 800, 1,000, 1,500). To the extent possible, compressed air and adhesive tape were employed to remove dust from cell lumina between each grit. This sample preparation protocol enabled the consistent and efficient preparation of wood samples for imaging.

**TABLE 1:** The seven xylaria providing images of wood specimens and the number of specimens from each collection used to build the training image data set.

**Institution (Xylarium acronym)**	**Specimen counts**
USDA Forest Products Laboratory, Madison collection (MADw)	501
USDA Forest Products Laboratory, Samuel J. Record collection (SJRw)	589
Instituto de Pesquisas Tecnologicas do Estado de Sao Paulo (BCTw)	139
Wood Laboratory, Universidad Distrital Francisco Jose de Caldas (BOFw)	37
Royal Museum of Central Africa (Tw)	32
Forestry Research Institute of Ghana (FORIGw)	2
**Mississippi State University (PACw)**	**119**

**The testing xylarium and specimen count is in bold case.**

### Image Collection

The XyloTron (Ravindran et al., 2020), an open-source macroscopic imaging system, was used to collect 6244 non-overlapping RGB images of the polished transverse surfaces of specimens from 228 taxa. Each XyloTron image shows 6.35 × 6.35 mm of tissue and has dimensions 2,048 × 2,048 pixels. Each institution employed one or more unique XyloTrons to collect images, so at least seven different hardware instantiations were employed. The details of the collected image dataset are presented in Table 2.

**TABLE 2:** Details of the image data set.

	**Training data (counts)**	**PACw data (counts)**	**Total (counts)**
Number of specimens	1,300	119	1,419
Number of images	5,715	529	6,244
Number of taxa	186	70	228*

1,419 specimens from 228 unique taxa were prepared and imaged. *Some taxa appeared in both data sets, thus the total number of taxa is not the sum of the training and testing taxa.

### Label Space Design

The 228 taxa included: (i) the species of interest to the Peruvian wood value chain, and (ii) additional congeneric macroscopically inseparable species native to South America. *Brosimum* was separated into two anatomically distinguishable classes while the remaining species were grouped into genus level classes, producing 24 classes. Complete details about the class labels and their constituent taxa are provided in Supplementary Material 2.

### Model Architecture and Training

**FIGURE 1:**
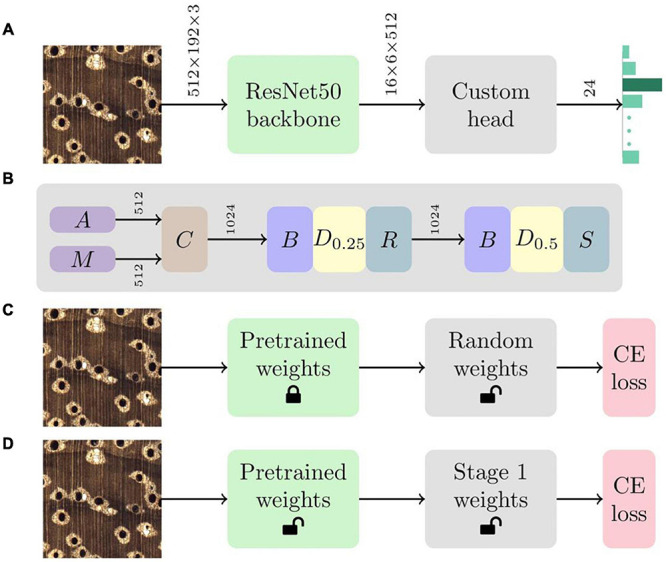
**(A)** The CNN architecture comprises a ResNet50 backbone with a custom head. Given an input image, the network produces a 24-element vector that represents the prediction confidence for each of the 24 classes in the model. Tensor dimensions are depicted over the connections between the modules. **(B)** The custom head includes global average pooling (A), global max pooling (M), concatenation (C), batchnorm (B), dropout (D) and linear layers with ReLU (R) and softmax (S) activations. D_*p*_ represents a dropout layer with drop probability parameter p. Tensor dimensions are depicted over the connections between the layers. **(C)** The first stage of transfer learning locks (or freezes) the ImageNet pretrained weights of the ResNet50 backbone and optimizes the randomly initialized weights of the custom head using the cross-entropy (CE) loss. **(D)** The weights of the entire network are fine tuned using the CE loss during the second stage of the training methodology.

A convolutional neural network (CNN; LeCun et al., 1989) classifier, with a aResNet50 (He et al., 2016) backbone and a custom head that included batchnorm (Ioffe and Szegedy, 2015), dropout (Srivastava et al., 2014), global average and max pooling, and linear layers (Goodfellow et al., 2016), was implemented for identification (see Figures 1A,B). A two-stage (Howard and Gugger, 2020) transfer learning (Pan and Yang, 2010) methodology, comprising locking the ImageNet (Russakovsky et al., 2015) pre-trained backbone weights while training the randomly initialized weights (He et al., 2015) of the custom head followed by fine tuning the weights of the entire network, was adopted (see Figures 1C,D). The Adam optimizer (Kingma and Ba, 2015) with simultaneous cosine annealing of the learning rate (maximum value of 1.8e-2) and momentum (Smith, 2018) was employed with cross-entropy loss for both the stages. Random 2,048 × 768 image patches were sampled from the training images, downsampled to 512 × 192 pixel images, and fed to the CNN in batches of size 16 with a data augmentation strategy that included horizontal/vertical flips, small rotations and cutout (Devries and Taylor, 2017). The hyperparameters were the same across all the training runs. Further details about the hyperparameter settings and training methodology can be found in Ravindran et al. (2020). The model definition, training and evaluation was performed using PyTorch (Paszke et al., 2019) and scientific Python tools (Pedregosa et al., 2011).

### Model Evaluation

The predictions of the trained models were evaluated at the specimen level with the predicted class for a specimen taken to be the majority of class predictions for the images contributed by the specimen. Accuracies based on the top prediction (top-1) for each specimen are reported for all the models. Additionally, the top two image-level predictions (from a specimen) are aggregated, with equal weights, to generate the top-2 predictions for a specimen. If the true label is one of the top-2 specimen level predictions, the specimen is considered to be correctly identified.

**FIGURE 2:**
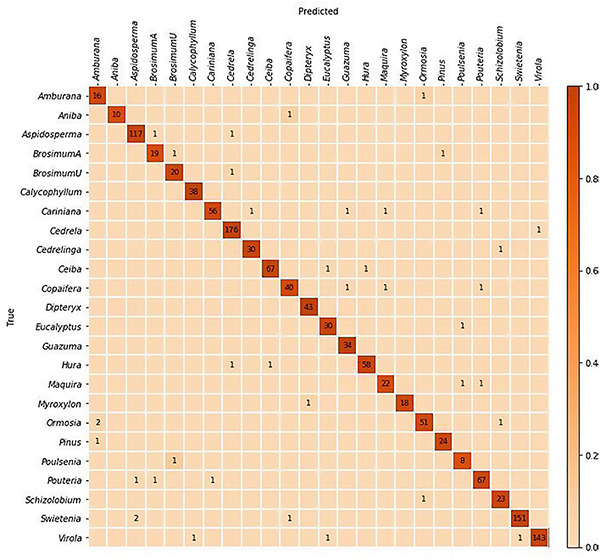
Confusion matrix for the top-1 predictions of the five-fold cross-validation models. The specimen-level accuracy accumulated over the five folds was 97%. The majority of misclassifications are between anatomically similar woods.

The following two analyses were performed to evaluate model performance in this study:

(1) Training and evaluation using five-fold cross validation: Images from 1,300 specimens were split into five folds with class level stratification while maintaining mutual exclusivity at the specimen level between the folds i.e., each specimen contributed images to exactly one fold. This specimen-aware partitioning of the data into folds with distinct specimens is required for correct evaluation of a trained model’s generalization capability to unseen samples. It should be noted that cross validation analysis did *not* include specimens from the PACw xylarium. A standard cross validation strategy, with four folds used for training and the trained model tested on the hold-out fold, was implemented and the specimen-level predictions over the five folds were accumulated to compute the accuracy (Table 3) and the confusion matrix (Figure 2).

**TABLE 3:** Predictive accuracies for the trained models and the corresponding number of specimen-level prediction errors.

	**Accuracy (%)**	**Number of specimens misclassified**
Predictions on cross-validation folds	97%	39/1,300
Top-1 prediction on PACw specimens	86.5%	16/119
Top-2 prediction on PACw specimens	92.4%	9/119

(2) Training a field model for evaluation on PACw specimens: All images in the five-fold cross-validation analysis were used to train a single model—the field model—using the same training hyperparameters. The specimen-level prediction performance of the field model was tested on 119 specimens from the PACw xylarium at Mississippi State University. The top-1 and top-2 predictions of the field model are reported in Table 3. The operators and XyloTron hardware used to collect the 529 images from the PACw specimens were different from those for the training data, and the images were used to evaluate the prediction accuracy of the trained model as a proxy for in-country field testing.

All images of the misclassified specimens in the five-fold cross validation were qualitatively evaluated and the misclassified specimens were categorized into three types: (1) taxa are anatomically consistent and the test specimen is typical; (2) the test specimen is atypical—but within reasonable variation for the taxon (i.e., it is not an archetypal image for the taxon); and (3) the taxa and test specimen are anatomically typical, but not anatomically consistent with each other. Types 1 and 2 represent misidentifications that trained field inspectors are likely to make, and so are sensible. Type 3 represents misidentifications not as likely to be made by trained human field inspectors, and for which there is no clear anatomical explanation.

## Results

The cross-validated specimen-level identification accuracy (accumulated over the five folds) was 97%. The field model had top-1 and top-2 specimen-level accuracies of 86.5 and 92.4% when tested on the PACw specimens. The predictive performance of the models is summarized in Table 3, and the cross-validation confusion matrix is shown in Figure 2.

**FIGURE 3:**
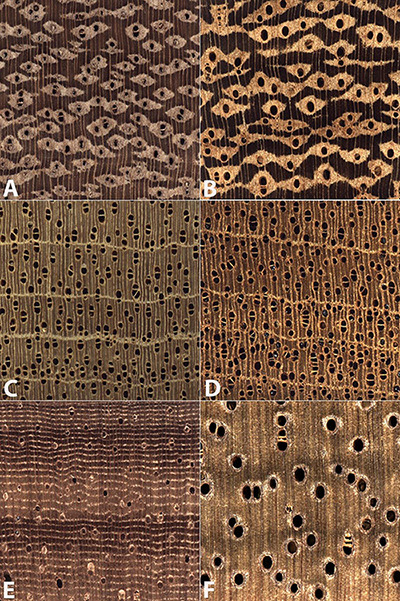
Images of the transverse surface of test specimens **(A,C,E)** and exemplars of the class to which they were assigned **(B,D,F)**. All images are 6.35 mm on a side. An anatomically representative specimen of class Amburana **(A)** was misclassified as the anatomically similar class Ormosia **(B)**. An anatomically atypical specimen of class Virola **(C)** was classified as class Swietenia **(D)**. An anatomically typical specimen of class Cariniana **(E)** was misclassified as the wood anatomically disparate class Cedrelinga **(F)**.

Figure 3 presents examples of each of the three types of misclassifications, which are summarized and reported in Table 4. When comparing two wood anatomically similar taxa (Type 1 misclassification, Figures 3A,B) the misclassification is sensible—both woods are characterized by vessels with similar grouping, arrangement, and of similar diameter, with lozenge-aliform-to-confluent axial parenchyma, and narrow, abundant rays. In Figures 3C,D (an example of Type 2 misclassification) the similarities between the atypical specimen of class Virola (*Virola surinamensis*; Figure 3C) and class Swietenia (*Swietenia macrophylla*; Figure 3D) include prominent marginal parenchyma, roughly similar vessel diameters, similar vessel grouping and arrangement, and absence of axial parenchyma in the body of the growth ring. An example of anatomically disparate misclassification (Type 3 misclassification) is shown in Figures 3E,F where the apotracheal banded parenchyma and much smaller vessels of class Cariniana (*Cariniana pyriformis*; Figure 3E) present a pattern not at all similar to the human eye to the larger vessels and vasicentric axial parenchyma of class Cedrelinga (*Cedrelinga cateniformis*; Figure 3F).

**TABLE 4:** Number and proportion of misclassified specimens from Figure 2 when categorizing into one of three misclassification types.

**Misclassification type**	**Number of misclassified specimens**	**Proportion of misclassified specimens**
Taxa are anatomically consistent, test specimen typical (Type 1)	13	0.333
Test specimen atypical for its taxon* (Type 2)	11	0.282
Taxa and test specimen are not anatomically consistent (Type 3)	15	0.385
Total	39	1.0

Types 1 and 2 are consistent with wood anatomy and the kind of misidentifications likely to be made by human field inspectors. Type 3 misclassifications are inconsistent with macroscopic wood anatomy and would not be expected to be made by a human inspector. *But within reasonable variation for the taxon.

## Discussion

The top-1 specimen-level accuracy of the field model was approximately 10 percentage points lower than the cross-validation accuracy while the top-2 specimen-level accuracy was over 90% — a level which is arguably sufficient to establish probable cause and initiate a full forensic investigation when fraud or misrepresentation is detected. The generalization capability of machine learning wood identification models must be evaluated on specimens that were not used to train the model. Additionally, real world systems deployed at scale must also be robust to the skills of operators (with different levels of training) and variations in system instantiations. The prediction accuracies reported above were obtained using training and testing datasets that were mutually exclusive at the specimen level. We maintained specimen-level mutual exclusivity of specimens across folds for cross validation analysis, and likewise xylaria specimen mutual exclusivity for field model evaluation. Additionally, the performance evaluation metrics were obtained using data collected at multiple sites and by multiple operators using different instantiations of the XyloTron system.

Our approach of testing models on specimens from a xylarium that did not contribute data to model training was employed as a logistically manageable, practically useful surrogate for real-world field testing. The ultimate test of any automated wood identification system is in-country field testing, but the main logistical challenge is the requirement of a wood identification expert for validation of the specimens being tested. Prior field-testing by Ravindran et al. (2019) of a pilot XyloTron model for Ghanaian woods showed a 10% drop in identification accuracy when comparing results on xylarium specimens to testing on field specimens. Such losses of accuracy of computer vision models when tested on wholly new datasets have been found by research in other domains of computer vision (Recht et al., 2018, 2019; Zech et al., 2018). The drop in performance shown in Ravindran et al. (2019) and in this study could be attributed to a combination of many factors such as differences in the quality of specimen surface preparation; differences in subtle anatomical patterns present in xylarium specimens as compared to material currently in trade; differences between green and dry wood; and slight variations in operator use of the equipment or the equipment itself. A well-designed field-testing strategy for evaluating automated wood identification systems must incorporate these factors in a context-specific manner. For example, given that the XyloTron platform is intended as a field-screening rather than a forensic tool, a testing protocol that incorporated taking multiple images per specimen of multiple specimens per shipment/consignment, etc., should yield reliable, robust results when characterizing the shipment at large, rather than any single piece of wood.

Our top-2 specimen-level accuracy was computed with equal weights for the top-2 image-level predictions, but for practical deployment a weighting scheme should be chosen in a context dependent fashion that takes into account factors such as the taxa-aware cost of making an incorrect identification, the anatomical similarity of the taxa being considered, the number of specimens to field screen per shipment, and the calibration of the model predictions (Niculescu-Mizil and Carauna, 2005; Guo et al., 2017). By including top-2 specimen level accuracy, we provide a window into the performance of the model and how such a model could be deployed. For example, the XyloTron platform’s classification software, *xyloinf* (Ravindran et al., 2020), provides the confidence value and an exemplar image for each class for the top-3 predictions per image, plus the sum of the confidences for the remaining N-3 classes in a given model of N classes. An operator thus has access not only to the ranked results, but also the confidence of a prediction and an exemplar image for human evaluation. This opens an interesting avenue for future research into the real-world deployment of computer vision wood identification systems (and other modalities) for maximum practical effect by incorporating human judgment (e.g., visual matching of an image from a field specimen to reference exemplar images for human approval and for flagging Type 3 misclassifications) or comparison of top-k results to some affirmative claim (e.g., a shipping manifest or transit permit). Even as field screening and forensic tools grow in power and sensitivity, it is critical to ensure that users of those tools are guided in how to achieve best practical effect with the tools at hand.

The uptake of computer vision and machine learning for automated wood identification is accelerating (Ravindran et al., 2018, 2019, 2020; de Andrade et al., 2020; Souza et al., 2020; Arévalo et al., 2021) and the real-world adoption of these systems is critically dependent on rigorous validation metrics and methodologies underlying any well-considered field-deployment framework. An easy first step toward rigorous validation is to enforce specimen-level separation between the training and testing splits (as in this work) rather than only image-level separation (most prior works). As affordable mobile phone adaptations (Tang et al., 2018; Wiedenhoeft, 2020) democratize access to these automated technologies, for wider impactful adoption it is critical that they be rigorously evaluated on external validation data. For this work, the next obvious steps will be testing the field model on specimens in Peruvian xylaria; folding in the PACw specimens to train a new field model to test in Peruvian xylaria; folding in the specimens from the Peruvian xylaria to iterate a new field model; and then, taking that model into the real-world and conducting the necessary field-testing coupled with independent forensic validation of the field tested specimens, an approach that should be applied to all modalities (Dormontt et al., 2015) in forensic wood science.

## Summary

We provided the largest tested computer vision wood identification model for Peruvian woods that is ready for immediate in-country field evaluation on the XyloTron platform. We demonstrated the utility and practicality of our model by evaluation using completely new specimens with independent hardware instantiations and different users, emphasized the critical need for specimen-level control of training and testing splits, and laid out a clear, iterative plan for augmenting the existing model. It is our hope that this work can be deployed within Peru to prevent illegally logged material from entering trade, and to support the trade in legal timber.

## Data Availability Statement

A minimal data set can be obtained by contacting the corresponding author and the trained models will be made available, but the full data set used in the study is protected for up to 5 years by a CRADA between FPL, UW-Madison, and FSC.

## Author Contributions

PV and RM provided the Peruvian timber market context and contributed the initial draft of the introduction. FO and RS provided access to and supervised data acquisition from the PACw test specimens. AWa prepared and imaged the PACw specimens. RM, FO, and AWi established the wood anatomical scope of the study. PR implemented the machine learning pipelines for the study. PR and AWi conducted data analysis, synthesis, and wrote the manuscript. All authors contributed to the article and approved the submitted version.

## Conflict of Interest

The authors declare that the research was conducted in the absence of any commercial or financial relationships that could be construed as a potential conflict of interest.

## References

[B1] Acevedo M. Kikata Y. (1994). *Atlas de Maderas del Peru.* Peru: Universidad Nacional Agraria La Molina.

[B2] Apolinario M. P. E. Huamán Bustamante S. G. Orellana G. C. (2018). “Deep learning applied to identification of commercial timber species from Peru,” in *Proceedings of the 2018 IEEE XXV International Conference on Electronics, Electrical Engineering and Computing (INTERCON)*, (Peru: IEEE).

[B3] Arévalo R. Pulido E. N. R. Solórzano J. F. G. Soares R. Ruffinatto F. Ravindran P. (2021). *Image based Identification of Colombian Timbers using the XyloTron: a Proof of Concept International Partnership (Identificación de Maderas Colombianas Utilizando el Xylotron: Prueba De Concepto de una Colaboración Internacional).* Colombia: Universidad Distrital Francisco Jose de Caldas.

[B4] Chavesta M. (2015). *Atlas Anatómico de Maderas del Perú II.* Lima: Universidad Nacional Agraria La Molina.

[B5] Chavesta M. (2018). *Atlas Anatómico de Maderas del Perú III.* Lima: Universidad Nacional Agraria La Molina.

[B6] de Andrade B. G. Basso V. M. de Figueiredo Latorraca J. V. (2020). Machine vision for field-level wood identification. *IAWA J.* 41 681–698. 10.1163/22941932-bja10001

[B7] Devries T. Taylor G. W. (2017). *Improved Regularization of Convolutional Neural Networks with Cutout.* New York: Cornell University

[B8] Dormontt E. E. Boner M. Braun B. Breulmann G. Degen B. Espinoza E. (2015). Forensic timber identification: it’s time to integrate disciplines to combat illegal logging. *Biol. Conserv.* 191 790–798. 10.1016/j.biocon.2015.06.038

[B9] Finer M. Jenkins C. N. Sky M. A. B. Pine J. (2014). Logging concessions enable illegal logging crisis in the Peruvian Amazon. *Sci. Rep.* 4:4719. 10.1038/srep04719 PMC5380163 24743552

[B10] Gasson P. (2011). How precise can wood identification be? Wood anatomy’s role in support of the legal timber trade, especially CITES. *IAWA J.* 32 137–154. 10.1163/22941932-90000049

[B11] Gontijo A. B. Rodrigues J. S. Santana C. S. Azevedo N. (2017). “have de Identificação “madeiras comerciais do Brasil” (Versão Android),” in *I Fórum de Anatomistas de Madeira da Amazônia (Famazon)* (Brazil: Desafios E Perspectivas Para Os Próximos Anos).

[B12] Gonzales E. V. (2008). *Identificación Organoléptica y Macroscópica de Maderas Comerciales.* CITEmadera: Lima.

[B13] Goodfellow I. Bengio Y. Courville A. (2016). *Deep Learning.* Cambridge, MA: The MIT Press.

[B14] Guo C. Pleiss G. Sun Y. Weinberger K. Q. (2017). “On calibration of modern neural networks,” in *Proceedings of the 34th International Conference on Machine Learning* (New York, NY: ACM).

[B15] He K. Zhang X. Ren S. Sun J. (2015). “Delving deep into rectifiers: surpassing human-level performance on ImageNet classification,” in *Proceedings of the 2015 International Conference on Computer Vision* (Santiago).

[B16] He K. Zhang X. Ren S. Sun J. (2016). “Deep residual learning for image recognition,” in *Proceedings of the 2016 IEEE Conference on Computer Vision and Pattern Recognition* (Las Vegas).

[B17] Howard J. Gugger S. (2020). Fastai: a layered API for deep learning. *Information* 11:108. 10.3390/info11020108

[B18] Ioffe S. Szegedy C. (2015). “Batch normalization: accelerating deep network training by reducing internal covariate shift,” in *Proceedings of the 32nd International Conference on Machine Learning* (France).

[B19] Khalid M. Lew E. Lee Y. Yusof R. Nadaraj M. (2008). Design of an intelligent wood species recognition system. *Int. J. Simulation Syst. Sci. Technol.* 9 9–19.

[B20] Kingma D. Ba J. (2015). “Adam: a method for stochastic optimization,” in *Proceedings of 2015 International Conference on Learning Representations* (San Diego).

[B21] Kometter R. (2019). *Asistencia Técnica para el Análisis del Funcionamiento del Modelo de Conceciones Forestales Maderables desde la Perspectiva Técnica. Evaluación del modelo de concesiones forestales con fines maderables, que compila los análisis legal, técnicoy económicofinanciero, así como las propuestas normativas para el fortalecimiento del modelo. PROYECTO.* Washington, DC: USAID.

[B22] LeCun Y. Boser B. Denker J. S. Henderson D. Howard R. E. Hubbard W. (1989). Backpropagation applied to handwritten zip code recognition. *Neural Comput.* 1 541–551. 10.1162/neco.1989.1.4.541

[B23] Niculescu-Mizil A. Carauna R. (2005). “Predicting good probabilities with supervised learning,” in *Proceedings of the 22nd International Conference on Machine Learning* (New York, NY: ACM).

[B24] Office of the US Trade Representative (2006). *United States - Peru Trade Promotion Agreement.* Washington, DC: Office of the US Trade Representative.

[B25] OSINFOR (2015). *onward. OSINFOR-SIGO. In: Supervision Agency for Wildlife Resources.* Available online at: https://observatorio.osinfor.gob.pe/Estadisticas/Home/Reportes/9. (Accessed September, 1, 2020).

[B26] OTCA (2018). *Clave de Identificación Electrónica de Especies Maderables de la Amazonia.* Peru: OCTA.

[B27] Pan S. J. Yang Q. (2010). A survey on transfer learning. *IEEE Trans. Knowledge Data Eng.* 22 1345–1359.

[B28] Paszke A. Gross S. Massa F. Lerer A. Bradbury J. Chanan G. (2019). Pytorch: an imperative style, high-performance deep learning library. *Adv. Neural Inform. Process. Syst.* 2019 8026–8037.

[B29] Pedregosa F. Varoquaux G. Gramfort A. Michel V. Thirion B. Grisel O. (2011). Scikit-learn: machine learning in Python. *J. Mach. Learn. Res.* 12 2825–2830.

[B30] Ravindran P. Wiedenhoeft A. C. (2020). Comparison of two forensic wood identification technologies for ten Meliaceae woods: computer vision versus mass spectrometry. *Wood Sci. Technol.* 46:1163.

[B31] Ravindran P. da Costa A. M. Soares R. Wiedenhoeft A. C. (2018). Classification of CITES-listed and other neotropical Meliaceae wood images using convolutional neural networks. *Plant Methods* 14:25. 10.1186/s13007-018-0292-9 PMC5865295 29588649

[B32] Ravindran P. Ebanyenle E. Ebeheakey A. A. Abban K. B. Lambog O. Soares R. (2019). “Image based identification of Ghanaian timbers using the XyloTron: opportunities, risks and challenges,” in *Proceedings 2019 Workshop on Machine Learning for the Developing World* (New York: Cornell University).

[B33] Ravindran P. Thompson B. J. Soares R. K. Wiedenhoeft A. C. (2020). The XyloTron: flexible, open-source, image-based macroscopic field identification of wood products. *Front. Plant Sci.* 11:1015. 10.3389/fpls.2020.01015 PMC7366520 32754178

[B34] Recht B. Roelofs R. Schmidt L. Shankar V. (2018). *Do CIFAR-10 Classifiers Generalize to CIFAR-10?.* New York: Cornell University.

[B35] Recht B. Roelofs R. Schmidt L. Shankar V. (2019). *Do ImageNet Classifiers Generalize to ImageNet?.* New York: Cornell University.

[B36] Rodriguez M. Sibille A. M. (1996). *Manual de Identificación de Especies Forestales de la Subregión Andina. Proyecto PD 150/91 Rev. I(I). “Identificación y Nomenclatura de las Maderas Tropicales Comerciales en la Subregión Andina.”.* Peru: ITTO.

[B37] Russakovsky O. Deng J. Su H. Krause J. Satheesh S. Ma S. (2015). Imagenet: large scale visual recognition challenge. *Int. J. Comput. Vis.* 115 211–252. 10.1007/s11263-015-0816-y

[B38] SBS and GIZ (2018). *Sectorial Assessment of Exposure to Money Laundering and Terrorist Financing Risks in the Peruvian Timber Sector.* Peru: Superintendency of Banking, Insurance and AFP.

[B39] SERFOR (2015). *Ley Forestal y de Fauna Silvestre N 29763 y sus Reglamentos. Bosques Productivos para la Vida.* Peru: SERFOR.

[B40] SERFOR (2020). Available online at: http://sniffs.serfor.gob.pe/estadistica/es

[B41] SERNANP (2020). *Nuestras Áreas Naturales Protegidas.* Peru: SERNANP.

[B42] Smith L. (2018). *A Disciplined Approach to Neural Network Hyper-Parameters: Part 1 – Learning Rate, Batch Size, Momentum, and Weight Decay.* New York: Cornell University.

[B43] Souza D. V. Santos J. X. Vieira H. C. Naide T. L. Nisgoski S. Oliveira L. E. S. (2020). An automatic recognition system of Brazilian flora species based on textural features of macroscopic images of wood. *Wood Sci. Technol.* 54 1065–1090. 10.1007/s00226-020-01196-z

[B44] Srivastava N. Hinton G. Krizhevsky A. Sutskever I. Salakhutdinov R. (2014). Dropout: a simple way to prevent neural networks from overfitting. *J. Mach. Learn. Res.* 15 1929–1958.

[B45] Tang X. J. Tay Y. H. Siam N. A. Lim S. C. (2018). “MyWood-ID: automated macroscopic wood identification system using smartphone and macro-lens,” in *Proceedings of the 2018 International Conference on Computational Intelligence and Intelligent Systems* (New York, NY: ACM).

[B46] Ugarte J. Mori I. (2018). *Guía Para la Identificación de la Madera de 50 Especies Forestales del Perú.* Peru: CITEmadera.

[B47] UNODC (2016). *Best Practice Guide for Forensic Timber Identification.* New York: UNDOC.

[B48] Wiedenhoeft A. C. (2020). The XyloPhone: toward democratizing access to high-quality macroscopic imaging for wood and other substrates. *IAWA J.* 41 699–719. 10.1163/22941932-bja10043

[B49] Zech J. R. Badgeley M. A. Liu M. Costa A. B. Titano J. J. Oermann E. K. (2018). Variable generalization performance of a deep learning model to detect pneumonia in chest radiographs: a cross-sectional study. *PLoS Med.* 15:e1002683. 10.1371/journal.pmed.1002683 30399157 PMC6219764

