# Supplementary material for: Field-Deployable Computer Vision Wood Identification of Peruvian Timbers
Source: Front Plant Sci. 2021 Jun 2;12:647515. doi: 10.3389/fpls.2021.647515 (PMC8206804; doi:10.3389/fpls.2021.647515)
Supplement: Supplementary file 2 [file Data_Sheet_2.pdf]

Supplement S2: Class composition details

Across the training and testing data sets a total of 188 taxa were partitioned into 24 classes based on wood anatomy similarities. For model training the taxa were grouped into genus level classes. It should be noted that some of the species included in the test dataset were not part of the training dataset. The species of the genus *Brosimum* were grouped into two classes: BrosimumA (anatomy similar to *Brosimum alicastrum*) and BrosimumU (anatomy similar to *Brosimum utile*).
In the table below, the column "Accession Taxon" lists the taxa of the specimen as they are recorded in the xylarium (and used for label space design) while the column "Verified Taxon" lists the taxa as verified using the online data base http://www.worldfloraonline.org/.
We would like to highlight and provide clarification on two specific cases of taxonomic flux encountered during the verification:

- The correct taxonomic designation of *Copaifera chodatiana* is *Guibortia chodatiana*. There was exactly **one** *Copaifera chodatiana* specimen that contributed **four** images to our data set. In our five-fold cross validation experiments, this specimen (when its images were part of the testing fold) was misclassified (both at top-1 and top-2 predictions), which is **the expected correct behavior**.
- Images from exactly one specimen of *Eucalyptus trachyphloia*, from the PACw xylarium, was included in our testing data set. The correct taxonomic designation of *Eucalyptus trachyphloia* is *Corymbia trachyphloia*. The wood anatomy of *Corymbia trachyphloia*, at the hand lens level, is consistent with that of *Eucalyptus*. This specimen was correctly identified by our model as class Eucalyptus at the top-2 prediction.

In conclusion, our label space design and accuracy results are both valid and consistent after accounting for taxonomic flux. The authors would like to gratefully acknowledge the reviewer who suggested accounting for taxonomic flux issues.

Table 1

Label	Accession Taxon	Verified Taxon	Train	Test
Amburana	*Amburana cearensis*	*Amburana cearensis*	✓	✓
Aniba	*Aniba bracteata*	*Aniba bracteata*	✓	
Aniba	*Aniba canelilla*	*Aniba canellila*	✓	
Aniba	*Aniba hostmanniana*	*Aniba hostmanniana*	✓	
Aniba	*Aniba nearparurensis*	*Aniba panurensis*		✓
Aniba	*Aniba riparia*	*Aniba riparia*	✓	
Aniba	*Aniba rosaeodora*	*Aniba rosaeodora*	✓	
Aniba	*Aniba trinitatis*	*Aniba citrifolia*	✓	
Aspidosperma	*Aspidosperma album*	*Aspidosperma album*	✓	
Aspidosperma	*Aspidosperma centrala*	*Aspidosperma album*	✓	
Aspidosperma	*Aspidosperma cruentum*	*Aspidosperma desmanthum*	✓	
Aspidosperma	*Aspidosperma cylindrocarpon*	*Aspidosperma cylindrocarpon*	✓	
Aspidosperma	*Aspidosperma desmanthum*	*Aspidosperma desmanthum*	✓	
Aspidosperma	*Aspidosperma macrocarpon*	*Aspidosperma macrocarpon*	✓	
Aspidosperma	*Aspidosperma marcgravianum*	*Aspidosperma excelsum*	✓	
Aspidosperma	*Aspidosperma megalocarpon*	*Aspidosperma megalocarpon*	✓	
Aspidosperma	*Aspidosperma melanocalix*	*Aspidosperma spruceanum*	✓	
Aspidosperma	*Aspidosperma parvifolium*	*Aspidosperma parvifolium*	✓	
Aspidosperma	*Aspidosperma polyneuron*	*Aspidosperma polyneuron*	✓	
Aspidosperma	*Aspidosperma rigidum*	*Aspidosperma rigidum*	✓	
Aspidosperma	*Aspidosperma* sp.	*Aspidosperma* sp.		✓
BrosimumA	*Brosimum alicastrum*	*Brosimum alicastrum*	✓	
BrosimumA	*Brosimum colimbianum*	*Brosimum alicastrum*		✓
BrosimumA	*Brosimum latifolium*	*Brosimum alicastrum*		✓
BrosimumA	*Brosimum terrabanum*	*Brosimum alicastrum*		✓
BrosimumU	*Brosimum utile*	*Brosimum utile*	✓	
Calycophyllum	*Calycophyllum acreanum*	*Calycophyllum megistocaulum*	✓	
Calycophyllum	*Calycophyllum candidissimum*	*Calycophyllum candidissimum*	✓	✓
Calycophyllum	*Calycophyllum multiflorum*	*Calycophyllum multiflorum*	✓	✓
Calycophyllum	*Calycophyllum obovatum*	*Calycophyllum obovatum*	✓	
Calycophyllum	*Calycophyllum spruceanum*	*Calycophyllum spruceanum*	✓	✓
Cariniana	*Cariniana domestica*	*Cariniana domestica*	✓	
Cariniana	*Cariniana estrellensis*	*Cariniana estrellensis*	✓	
Cariniana	*Cariniana excelsa*	*Cariniana estrellensis*	✓	
Cariniana	*Cariniana exigua*	*Cariniana pyriformis*		✓
Cariniana	*Cariniana legalis*	*Cariniana legalis*	✓	✓
Cariniana	*Cariniana micrantha*	*Cariniana micrantha*	✓	
Cariniana	*Cariniana pyriformis*	*Cariniana pyriformis*	✓	✓
Cedrela	*Cedrela angustifolia*	*Cedrela angustifolia*	✓	
Cedrela	*Cedrela fissilis*	*Cedrela fissilis*	✓	✓
Cedrela	*Cedrela lilloi*	*Cedrela angustifolia*	✓	
Cedrela	*Cedrela montana*	*Cedrela montana*	✓	

Table 1 (continued)

Label	Accession Taxon	Verified Taxon	Train	Test
Cedrela	*Cedrela odorata*	*Cedrela odorata*	✓	✓
Cedrela	*Cedrela rosei*	*Cedrela rosei*		✓
Cedrela	*Cedrela* sp.	*Cedrela* sp.		✓
Cedrela	*Cedrela whitfordii*	*Cedrela odorata*		✓
Cedrelinga	*Cedrelinga cateniformis*	*Cedrelinga cateniformis*	✓	
Ceiba	*Ceiba pentandra*	*Ceiba pentandra*	✓	
Ceiba	*Ceiba samauma*	*Ceiba samauma*	✓	
Ceiba	*Ceiba speciosa*	*Ceiba speciosa*	✓	
Ceiba	*Chorisia insignis*	*Ceiba insignis*	✓	
Ceiba	*Chorisia integrifolia*	*Ceiba insignis*	✓	
Ceiba	*Chorisia speciosa*	*Ceiba speciosa*	✓	
Copaifera	*Copaifera aromatica*	*Copaifera aromatica*	✓	
Copaifera	*Copaifera canime*	*Copaifera canime*	✓	
Copaifera	*Copaifera chiriquensis*	*Copaifera* sp.	✓	
Copaifera	*Copaifera chodatiana*	*Guibourtia chodatiana*	✓	
Copaifera	*Copaifera guianensis*	*Copaifera guyanensis*	✓	
Copaifera	*Copaifera langsdorffii*	*Copaifera langsdorffii*	✓	
Copaifera	*Copaifera langsdorfii*	*Copaifera langsdorffii*	✓	
Copaifera	*Copaifera majorina*	*Copaifera majorina*	✓	
Copaifera	*Copaifera martii*	*Copaifera martii*	✓	
Copaifera	*Copaifera multijuga*	*Copaifera multijuga*	✓	
Copaifera	*Copaifera officinalis*	*Copaifera officinalis*	✓	
Copaifera	*Copaifera panamensis*	*Copaifera panamensis*	✓	
Copaifera	*Copaifera religiosa*	*Copaifera religiosa*	✓	
Copaifera	*Copaifera reticulata*	*Copaifera reticulata*	✓	✓
Copaifera	*Copaifera trapezifolia*	*Copaifera trapezifolia*	✓	
Dipteryx	*Dipteryx alata*	*Dipteryx alata*	✓	
Dipteryx	*Dipteryx cf-ferrea*	*Dipteryx micrantha*	✓	
Dipteryx	*Dipteryx magnifica*	*Dipteryx magnifica*	✓	
Dipteryx	*Dipteryx micrantha*	*Dipteryx micrantha*	✓	
Dipteryx	*Dipteryx odorata*	*Dipteryx odorata*	✓	✓
Dipteryx	*Dipteryx oleifera*	*Dipteryx oleifera*	✓	
Dipteryx	*Dipteryx polyphylla*	*Dipteryx polyphylla*	✓	
Dipteryx	*Dipteryx punctata*	*Dipteryx punctata*	✓	
Dipteryx	*Dipteryx rosea*	*Dipteryx rosea*	✓	
Dipteryx	*Dipteryx* sp.	*Dipteryx* sp.	✓	
Eucalyptus	*Eucalyptus acmenioides*	*Eucalyptus acmenoides*		✓
Eucalyptus	*Eucalyptus botryoides*	*Eucalyptus botryoides*		✓
Eucalyptus	*Eucalyptus camaldulensis*	*Eucalyptus camaldulensis*	✓	
Eucalyptus	*Eucalyptus capitellata*	*Eucalyptus capitellata*		✓
Eucalyptus	*Eucalyptus cornuta*	*Eucalyptus cornuta*		✓
Eucalyptus	*Eucalyptus dawsonii*	*Eucalyptus dawsonii*		✓
Eucalyptus	*Eucalyptus deglupta*	*Eucalyptus deglupta*	✓	✓

Table 1 (continued)

Label	Accession Taxon	Verified Taxon	Train	Test
Eucalyptus	*Eucalyptus delegatensis*	*Eucalyptus delegatensis*		✓
Eucalyptus	*Eucalyptus diversicolor*	*Eucalyptus diversicolor*		✓
Eucalyptus	*Eucalyptus eugenoides*	*Eucalyptus eugenioides*		✓
Eucalyptus	*Eucalyptus gigantea*	*Eucalyptus globulus*		✓
Eucalyptus	*Eucalyptus gomphocephala*	*Eucalyptus gomphocephala*		✓
Eucalyptus	*Eucalyptus grandis*	*Eucalyptus grandis*	✓	
Eucalyptus	*Eucalyptus hemiphloia*	*Eucalyptus moluccana*		✓
Eucalyptus	*Eucalyptus longifolia*	*Eucalyptus elata*		✓
Eucalyptus	*Eucalyptus macrorhyncha*	*Eucalyptus macrorhyncha*		✓
Eucalyptus	*Eucalyptus maculata*	*Corymbia maculata*		✓
Eucalyptus	*Eucalyptus marginata*	*Eucalyptus marginata*	✓	✓
Eucalyptus	*Eucalyptus muelleriana*	*Eucalyptus muelleriana*		✓
Eucalyptus	*Eucalyptus obliqua*	*Eucalyptus obliqua*	✓	✓
Eucalyptus	*Eucalyptus pilularis*	*Eucalyptus pilularis*	✓	✓
Eucalyptus	*Eucalyptus propinqua*	*Eucalyptus propinqua*		✓
Eucalyptus	*Eucalyptus redunea*	*Eucalyptus redunca*		✓
Eucalyptus	*Eucalyptus regnans*	*Eucalyptus regnans*	✓	
Eucalyptus	*Eucalyptus robusta*	*Eucalyptus robusta*	✓	
Eucalyptus	*Eucalyptus rostrata*	*Eucalyptus camaldulensis*		✓
Eucalyptus	*Eucalyptus rudderi*	*Eucalyptus rudderi*		✓
Eucalyptus	*Eucalyptus saligna*	*Eucalyptus saligna*	✓	✓
Eucalyptus	*Eucalyptus siderophoboia*	*Eucalyptus siderophloia*		✓
Eucalyptus	*Eucalyptus sieberiana*	*Eucalyptus sieberi*		✓
Eucalyptus	*Eucalyptus tereticornis*	*Eucalyptus tereticornis*		✓
Eucalyptus	*Eucalyptus trachyphloia*	*Corymbia trachyphloia*		✓
Guazuma	*Guazuma crinita*	*Guazuma crinita*	✓	
Guazuma	*Guazuma guazuma*	*Guazuma ulmifolia*	✓	
Guazuma	*Guazuma rosea*	*Guazuma crinita*	✓	
Guazuma	*Guazuma* sp.	*Guazuma* sp.	✓	
Guazuma	*Guazuma tomentosa*	*Guazuma ulmifolia*	✓	
Guazuma	*Guazuma ulmifolia*	*Guazuma ulmifolia*	✓	
Hura	*Hura crepitans*	*Hura crepitans*	✓	
Maquira	*Maquira calophylla*	*Maquira calophylla*	✓	
Maquira	*Maquira coriacea*	*Maquira coriacea*	✓	
Maquira	*Maquira costaricana*	*Maquira guianensis*	✓	
Maquira	*Maquira guianensis*	*Maquira guianensis*	✓	
Maquira	*Maquira sclerophylla*	*Maquira sclerophylla*	✓	
Maquira	*Maquira* sp.	*Maquira* sp.	✓	
Myroxylon	*Myroxylon balsamum*	*Myroxylon balsamum*	✓	
Myroxylon	*Myroxylon balsamun*	*Myroxylon balsamum*		✓
Myroxylon	*Myroxylon peruiferum*	*Myroxylon peruiferum*	✓	
Myroxylon	*Myroxylon toluiferum*	*Myroxylon balsamum*	✓	
Ormosia	*Ormosia amazonica*	*Ormosia amazonica*	✓	

Table 1 (continued)

Label	Accession Taxon	Verified Taxon	Train	Test
Ormosia	*Ormosia avilensis*	*Ormosia avilensis*	✓	
Ormosia	*Ormosia coccinea*	*Ormosia coccinea*	✓	✓
Ormosia	*Ormosia colombiana*	*Gongylolepis colombiana*	✓	
Ormosia	*Ormosia costulata*	*Ormosia costulata*	✓	
Ormosia	*Ormosia coutinhoi*	*Ormosia coutinhoi*	✓	
Ormosia	*Ormosia dasycarpa*	*Ormosia monosperma*	✓	
Ormosia	*Ormosia elata*	*Ormosia elata*	✓	
Ormosia	*Ormosia excelsa*	*Ormosia excelsa*	✓	✓
Ormosia	*Ormosia flava*	*Ormosia flava*	✓	✓
Ormosia	*Ormosia isthmensis*	*Ormosia isthmensis*	✓	
Ormosia	*Ormosia krugii*	*Ormosia krugii*	✓	
Ormosia	*Ormosia larecajana*	*Ormosia larecajana*	✓	
Ormosia	*Ormosia lignivalis*	*Ormosia lignivalvis*	✓	
Ormosia	*Ormosia macrocalyx*	*Ormosia macrocalyx*	✓	
Ormosia	*Ormosia monosperma*	*Ormosia monosperma*	✓	
Ormosia	*Ormosia nobilis*	*Ormosia nobilis*	✓	
Ormosia	*Ormosia pacycarpa*	*Ormosia pachycarpa*		✓
Ormosia	*Ormosia panamensis*	*Ormosia panamensis*	✓	✓
Ormosia	*Ormosia paraensis*	*Ormosia paraensis*	✓	
Ormosia	*Ormosia smithii*	*Ormosia smithii*	✓	
Ormosia	*Ormosia stipularis*	*Ormosia stipularis*	✓	
Ormosia	*Ormosia toledoana*	*Ormosia macrocalyx*	✓	
Pinus	*Pinus ayacahuite*	*Pinus ayacahuite*	✓	
Pinus	*Pinus caribaea*	*Pinus caribaea*	✓	
Pinus	*Pinus hartwegii*	*Pinus hartwegii*	✓	
Pinus	*Pinus montezumae*	*Pinus hartwegii*	✓	
Pinus	*Pinus occidentalis*	*Pinus montezumae*	✓	✓
Pinus	*Pinus oocarpa*	*Pinus oocarpa*	✓	
Pinus	*Pinus patula*	*Pinus patula*	✓	
Pinus	*Pinus pseudostrobus*	*Pinus montezumae*	✓	
Pinus	*Pinus tropicalis*	*Pinus tropicalis*	✓	
Poulsenia	*Poulsenia armata*	*Poulsenia armata*	✓	✓
Pouteria	*Pouteria aff-gongrijpii*	*Pouteria gongrijpii*	✓	
Pouteria	*Pouteria aff-reticulata*	*Planchonella reticulata*	✓	
Pouteria	*Pouteria bilocularis*	*Pouteria bilocularis*	✓	
Pouteria	*Pouteria caimito*	*Pouteria caimito*	✓	
Pouteria	*Pouteria cuspidata*	*Pouteria cuspidata*	✓	
Pouteria	*Pouteria decorticans*	*Pouteria decorticans*	✓	
Pouteria	*Pouteria engleri*	*Pouteria engleri*	✓	
Pouteria	*Pouteria filipes*	*Pouteria filipes*	✓	
Pouteria	*Pouteria furcata*	*Pouteria furcata*	✓	
Pouteria	*Pouteria glomerata*	*Pouteria glomerata*	✓	
Pouteria	*Pouteria gomphiifolia*	*Pouteria gomphiifolia*	✓	

Table 1 (continued)

Label	Accession Taxon	Verified Taxon	Train	Test
Pouteria	*Pouteria guianensis*	*Pouteria guianensis*	✓	
Pouteria	*Pouteria gutta*	*Pouteria torta*	✓	
Pouteria	*Pouteria hispida*	*Pouteria hispida*	✓	
Pouteria	*Pouteria jariensis*	*Pouteria jariensis*	✓	
Pouteria	*Pouteria krukovii*	*Pouteria krukovii*	✓	
Pouteria	*Pouteria lasiocarpa*	*Pouteria caimito*	✓	
Pouteria	*Pouteria laurifolia*	*Pouteria caimito*	✓	
Pouteria	*Pouteria macrocarpa*	*Pouteria multiflora*	✓	
Pouteria	*Pouteria macrophylla*	*Pouteria macrophylla*	✓	
Pouteria	*Pouteria multiflora*	*Pouteria multiflora*		✓
Pouteria	*Pouteria ovata*	*Pouteria ramiflora*	✓	
Pouteria	*Pouteria plicata*	*Pouteria plicata*	✓	
Pouteria	*Pouteria procera*	*Pouteria procera*	✓	
Pouteria	*Pouteria ramiflora*	*Pouteria ramiflora*	✓	
Pouteria	*Pouteria reticulata*	*Pouteria reticulata*	✓	
Pouteria	*Pouteria salicifolia*	*Pouteria salicifolia*	✓	
Pouteria	*Pouteria solimoesensis*	*Pouteria hispida*	✓	
Pouteria	*Pouteria surumuensis*	*Pouteria surumuensis*	✓	
Pouteria	*Pouteria torta*	*Pouteria torta*	✓	
Pouteria	*Pouteria torta-glabra*	*Pouteria torta*	✓	
Pouteria	*Pouteria trichopoda*	*Pouteria hispida*	✓	
Pouteria	*Pouteria trilocularis*	*Pouteria trilocularis*	✓	
Pouteria	*Pouteria vestita*	*Sarcaulus vestitus*	✓	
Schizolobium	*Schizolobium amazonicum*	*Schizolobium amazonicum*	✓	
Schizolobium	*Schizolobium excelsum*	*Schizolobium parahyba*		✓
Schizolobium	*Schizolobium parahyba*	*Schizolobium parahyba*	✓	
Schizolobium	*Schizolobium parahybum*	*Schizolobium parahyba*		✓
Schizolobuim	*Schizolobuim excelsum*	*Schizolobium parahyba*		✓
Swietenia	*Swietenia macrophylla*	*Swietenia macrophylla*	✓	✓
Swietenia	*Swietenia* sp.	*Swietenia* sp.		✓
Virola	*Virola bicuhyba*	*Virola bicuhyba*	✓	
Virola	*Virola calophylla*	*Virola calophylla*	✓	✓
Virola	*Virola calophylloidea*	*Virola calophylloidea*	✓	
Virola	*Virola carinata*	*Virola carinata*	✓	
Virola	*Virola cuspidata*	*Virola elongata*		✓
Virola	*Virola elongata*	*Virola elongata*	✓	
Virola	*Virola flexuosa*	*Virola flexuosa*	✓	
Virola	*Virola gardneri*	*Virola gardneri*	✓	
Virola	*Virola gracilis*	*Virola surinamensis*	✓	
Virola	*Virola guatemalensis*	*Virola guatemalensis*	✓	
Virola	*Virola koschnyi*	*Virola koschnyi*	✓	
Virola	*Virola loretensis*	*Virola loretensis*	✓	✓
Virola	*Virola macrocarpa*	*Virola macrocarpa*	✓	

Table 1 (continued)

Label	Accession Taxon	Verified Taxon	Train	Test
Virola	*Virola melinonii*	*Virola michelii*	✓	
Virola	*Virola merendonia*	*Virola koschnyi*		✓
Virola	*Virola michelii*	*Virola michelii*	✓	
Virola	*Virola molissima*	*Virola mollissima*	✓	✓
Virola	*Virola multicostata*	*Virola multicostata*	✓	
Virola	*Virola multinervia*	*Virola multinervia*	✓	
Virola	*Virola officinalis*	*Virola officinalis*	✓	✓
Virola	*Virola oleifera*	*Bicuiba oleifera*	✓	
Virola	*Virola pavonis*	*Virola pavonis*	✓	
Virola	*Virola sebifera*	*Virola sebifera*	✓	✓
Virola	*Virola* sp.	*Virola* sp.	✓	
Virola	*Virola surinamensis*	*Virola surinamensis*	✓	✓
Virola	*Virola uaupensis*	*Virola elongata*		✓
Virola	*Virola venosa*	*Virola venosa*	✓	

Table 1: The class labels and their constituent taxa.
